# Purine metabolism rewiring improves glioblastoma susceptibility to temozolomide treatment

**DOI:** 10.1038/s41419-025-07667-0

**Published:** 2025-04-24

**Authors:** Simona D’Aprile, Simona Denaro, Filippo Torrisi, Lucia Longhitano, Sebastiano Giallongo, Cesarina Giallongo, Vittorio Bontempi, Claudio Bucolo, Filippo Drago, Maria Caterina Mione, Giovanni Li Volti, Maja Potokar, Jernej Jorgačevski, Robert Zorec, Daniele Tibullo, Angela Maria Amorini, Nunzio Vicario, Rosalba Parenti

**Affiliations:** 1 Department of Biomedical and Biotechnological Sciences, University of Catania, Catania, Italy; 2 Department of Medicine and Surgery, University of Enna “Kore”, Enna, Italy; 3 Department of Drug and Health Sciences, University of Catania, Catania, Italy; 4 Department of Medical and Surgical Sciences and Advanced Technologies, F. Ingrassia, University of Catania, Catania, Italy; 5 Department of Cellular, Computational and Integrative Biology (CIBIO) and Centre of Medical Sciences (CISMed), University of Trento, Trento, Italy; 6 Laboratory of Neuroendocrinology‑Molecular Cell Physiology, Institute of Pathophysiology, Faculty of Medicine, University of Ljubljana, Ljubljana, Slovenia; 7 Celica Biomedical, Ljubljana, Slovenia

**Keywords:** Biochemistry, Diseases of the nervous system

## Abstract

Glioblastoma (GBM) is among the deadliest cancers, characterized by poor prognosis and median survival of 12–15 months post-diagnosis. Despite aggressive therapeutic regimens, GBM treatment is still an unmet clinical need due to heterogeneity, recurrencies, and resistance. Metabolic reshaping is emerging as a critical mechanism supporting cell proliferation and sustaining chemoresistance. In this study, we explored metabolic changes induced by chemotherapy in temozolomide (TMZ)-sensitive and TMZ-resistant GBM cell lines. We found that purine levels were altered in sensitive versus resistant GBM cells, highlighting a critical role of guanosine and inosine metabolism. By using a mesenchymal-like GBM zebrafish model, we uncovered dysregulated pathways involved in purine metabolism, with a downregulation of catabolic processes. Our data indicate that combined treatment with TMZ plus guanosine and inosine increased cytotoxicity, enhancing chemotherapy effectiveness in TMZ-resistant cells. These effects correlated with alterations in mitochondrial dynamics and activity. Specifically, the combinatorial effectiveness of TMZ with guanosine and inosine was linked to Mitofusin-2 overexpression, enhancing mitochondrial fusion, typically associated with a better prognosis. Therefore, our findings suggest that purine metabolism is involved in the metabolic rewiring of TMZ-resistant cells, suggesting guanosine and inosine as potential adjuvant treatments to improve the cytotoxicity effects of chemotherapy in resistant GBM.

## Introduction

Glioblastoma (GBM) is a highly malignant and deadly brain tumour characterised by a poor prognosis and a median survival of 12–15 months post-diagnosis [1]. To date, in most cases, GBM has shown resistance to conventional treatments, including radiotherapy and chemotherapy, which together with surgical resection represent the gold standard approach [2]. In particular, GBM heterogeneity and resistance strongly limit therapeutic effectiveness [3]. Histologically, GBM shows significant differences between patients, and this diversity is confirmed at a molecular level by a wide range of possible genetic alterations [4]. Based on molecular features, a genomic analysis has grouped GBM into three subclasses: classical, mesenchymal and proneural, showing distinctive features and a wide degree of sensitivity to therapy [5]. Typically, mesenchymal tumours are aggressive and more resistant to therapy, while proneural ones are relatively sensitive to treatments and less infiltrative [6, 7]. In addition, during tumour progression, GBM could shift from a proneural to a mesenchymal subtype, leading to chemoresistance [8]. In particular, Tumour Associated Macrophages (TAMs), through the secretion of anti-inflammatory cytokines, support the GBM subtype transition, driving the tumour to adopt a mesenchymal profile [9].

The standard-of-care chemotherapy for GBM patients includes temozolomide (TMZ), an antineoplastic alkylating agent of DNA. Throughout its lipophilicity, size and stability at low pH, TMZ can cross the blood-brain barrier, acting on the central nervous system (CNS) tumours [10, 11]. However, GBM can employ different strategies to resist TMZ, primarily including DNA repair with O6-methylguanine-DNA methyltransferase (MGMT) activation, drug efflux through ATP-binding cassette (ABC) transporters, epigenetic modifications and metabolic rewiring [12]. Altered metabolic pathways are a typical hallmark of GBM, which can reshape its metabolism depending on nutrient availability. Metabolic reprogramming increases GBM invasiveness and aggressiveness, supporting tumour growth also in a hypoxic environment [13]. It is well known that GBM takes advantage of glycolysis to increase its proliferation rate by metabolising glucose into lactate, even in a normoxic environment, leading to the Warburg effect [14, 15]. Beyond an improved glycolysis, GBM can modulate lipid and nucleotide metabolism to enhance de novo lipid and nucleotide synthesis, thus adapting to increased energy requests [16]. However metabolic reshaping is also one of the key mechanisms to overcome cytotoxicity of standard therapy. Through metabolic reprogramming, GBM cells decrease cellular stress caused by radio- and chemo-therapy, leading to therapeutic tolerance and supporting tumour growth [17].

Moreover, recent studies show that tumour genotype and microenvironment affect metabolic rewiring in GBM cells, inducing alterations that may play a role in developing novel therapeutic strategies [15]. Growing evidence demonstrates that drugs targeting cell metabolism in combination with chemotherapy or radiotherapy could increase the effectiveness of anti-cancer approaches [18]. Specifically, metabolomic analysis allows the study of tumour metabolic reprogramming, investigating the molecular pathways involved in GBM metabolic alterations, to discover new therapeutic targets [19]. However, highlighting metabolic targets for GBM therapy is challenging due to its heterogeneity; thus, it is crucial to characterise GBM from a metabolic perspective [20].

Herein, we aimed to investigate GBM metabolic rewiring by combining both in vitro and in vivo approaches. Particularly, we focused on the study of metabolic alterations after chemotherapy treatment in different GBM cell lines: U-251 MG of proneural subtype sensitive to TMZ, T98-G of mesenchymal subtype resistant to TMZ, and U-251 MG R, induced to develop TMZ resistance. Our results revealed that purine metabolism plays a crucial role in cellular mechanisms leading to TMZ resistance. Our findings on GBM cell lines were confirmed in an in vivo zebrafish model of mesenchymal GBM, showing dysregulated pathways involving purines, thus suggesting a role of metabolic reshaping in TMZ susceptibility. We found that mitochondrial fitness and dynamics were affected by the increased levels of guanosine and inosine in resistant cells and these changes were positively correlated with mitochondrial fusion.

## Results

### U-251 MG R and T98-G cells exhibit resistance to TMZ

**Fig. 1:**
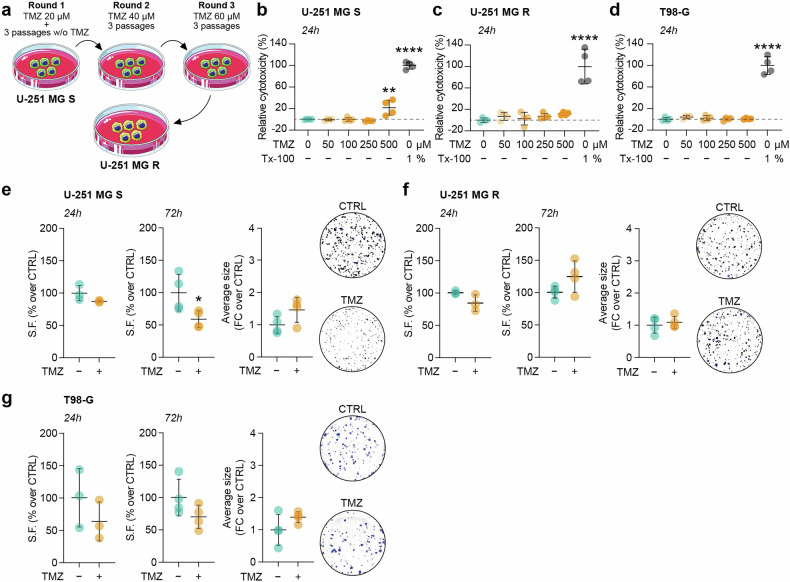
U-251 MG S, U-251 MG R and T98-G cells differently tolerate TMZ. **a** Schematic representation of the protocol used to induce TMZ resistance in U-251 MG S cells. **b**–**d** LDH assay at 24 h on U-251 MG S (**b**), U-251 MG R (**c**) and T98-G (**d**) cells treated with 0, 50, 100, 250, 500 μM of TMZ. **e**–**g** Surviving fraction (SF) at 24 h and 72 h post-treatment, quantification of average clones size and representative pictures of the clonogenic assay at 72 h of U-251 MG S (**e**), U-251 MG R (**f**) and T98-G (**g**) clonogenic assay. Data are shown via scattered dot plots as mean ± SD of n ≥ 3 independent experiments. *p-value < 0.05; **p-value < 0.01; **** p-value < 0.0001. CTRL control, FC fold change, SF surviving fraction, TMZ temozolomide, Tx-100 Triton X-100.

U-251 MG S, spontaneously sensitive to TMZ, were used to generate a TMZ resistant-induced cell line (i.e. U-251 MG R) following the protocol schematized in Fig. 1a, to obtain a GBM cell line with intermediate characteristics suitable for studying the mechanisms underlying chemotherapy resistance. To test cells sensitivity to chemotherapy, we analysed TMZ-induced cytotoxic effects on U-251 MG S, U-251 MG R and T98-G cells, spontaneously resistant to TMZ, evaluating relative cytotoxicity at 24, 48, 72 h post-TMZ administration at different concentrations (50, 100, 250, 500 μM) (Figs. 1b–d and S1). Data showed that at 24 h, U-251 MG S cells exhibited a significant increase in relative cytotoxicity to 500 μM TMZ, while no effects were observed in U-251 MG R and T98-G cells at any tested concentrations (Fig. 1b–d). These results were confirmed at 48 and 72 h, proving that relative cytotoxicity (%) increased at 250 and 500 μM of TMZ in U-251 MG S cells, while changes were observed in U-251 MG R cells at 48 h and 72 h only at the dose of 500 μM. Additionally, T98-G cells showed an effect only at 72 h with the highest tested concentration (Fig. S1).

We also performed a clonogenic assay to assess the effect of TMZ on cell proliferation, demonstrating a decrease in the surviving fraction of U-251 MG S cells following 72 h treatment with TMZ, whereas no significant effects on U-251 MG R and T98-G cells were observed (Fig. 1e–g). We did not observe significant changes in the average size of clones in all tested cell lines (Fig. 1e–g).

Therefore, our results confirmed that U-251 MG S cells were sensitive to TMZ, while T98-G cells showed resistance to the drug. Notably, U-251 MG R cells demonstrated a resistance profile comparable to the spontaneously resistant T98-G cells.

### Sensitive and resistant cell lines show different metabolic profiles

**Fig. 2:**
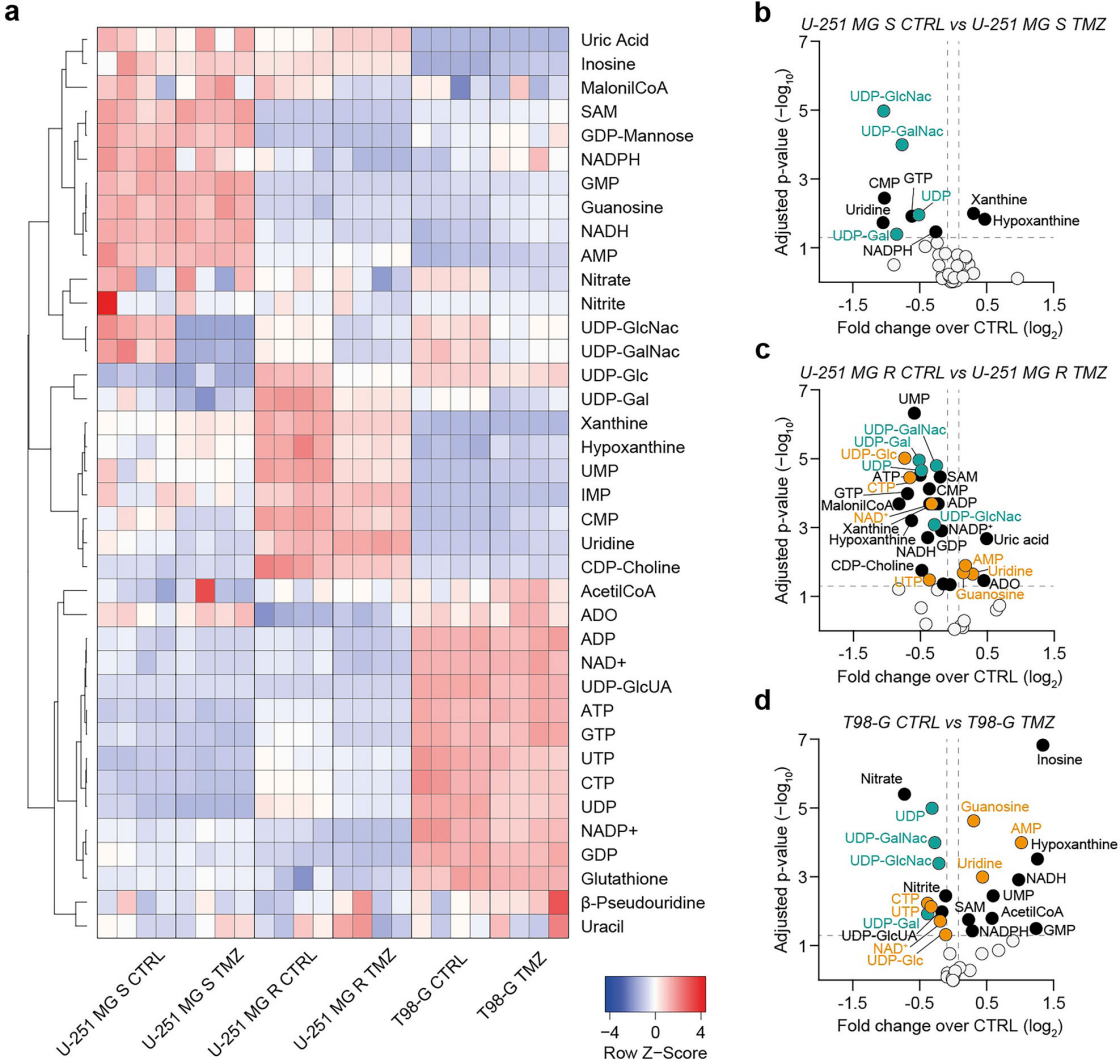
Sensitive and resistant GBM cells display distinct metabolic profiles. **a** Heatmap of Z-Score values based on the abundance of 38 metabolites. **b**–**d** Volcano plots of metabolites levels expressed as log_2_ fold changes over CTRL and −log_10_ of adjusted p-value in U-251 MG S CTRL versus TMZ-treated (**b**), U-251 MG R CTRL versus TMZ-treated (**c**), T98-G CTRL versus TMZ-treated (**d**). White dots represent not significantly modulated metabolites; black dots represent significantly modulated metabolites; green dots represent metabolites showing the same trend in all tested cell lines; orange dots represent metabolites showing the same trend in resistant cell lines (i.e. U-251 MG R and T98-G). Data include n = 4 independent replicates per group. CTRL control, TMZ temozolomide.

Our next aim was to investigate metabolic changes involved in chemotherapy resistance. Thus, to study metabolic profiles in sensitive and resistant cells, we assessed a targeted metabolomic analysis on the 3 GBM cell lines. These results revealed different levels of specific metabolites among the 3 tested cell lines, with U-251 MG R cells showing an intermediate metabolic profile between those of U-251 MG S and T98-G cells (Fig. 2). We observed a better antioxidant and energetic metabolic profile in T98-G cells as compared to U-251 MG cell lines, as demonstrated by the higher levels of energetic phosphates, such as adenosine triphosphate (ATP), guanosine triphosphate (GTP), and of glutathione (GSH; Fig. 2a). Furthermore, through volcano plots analysis of CTRL *vs* TMZ treatment, we evaluated metabolites that changed in the same direction across all tested cell lines (green dots) or specifically among resistant cells (orange dots), following TMZ treatment (Fig. 2b–d). These data revealed a similar pattern involving uridine diphosphate (UDP) derivatives upon TMZ treatment in all tested cell lines, while most of the metabolites shared among resistant cells were associated with nucleotide metabolism (Fig. 2b–d).

### Guanosine and inosine increase chemotherapy efficacy in resistant cells

**Fig. 3:**
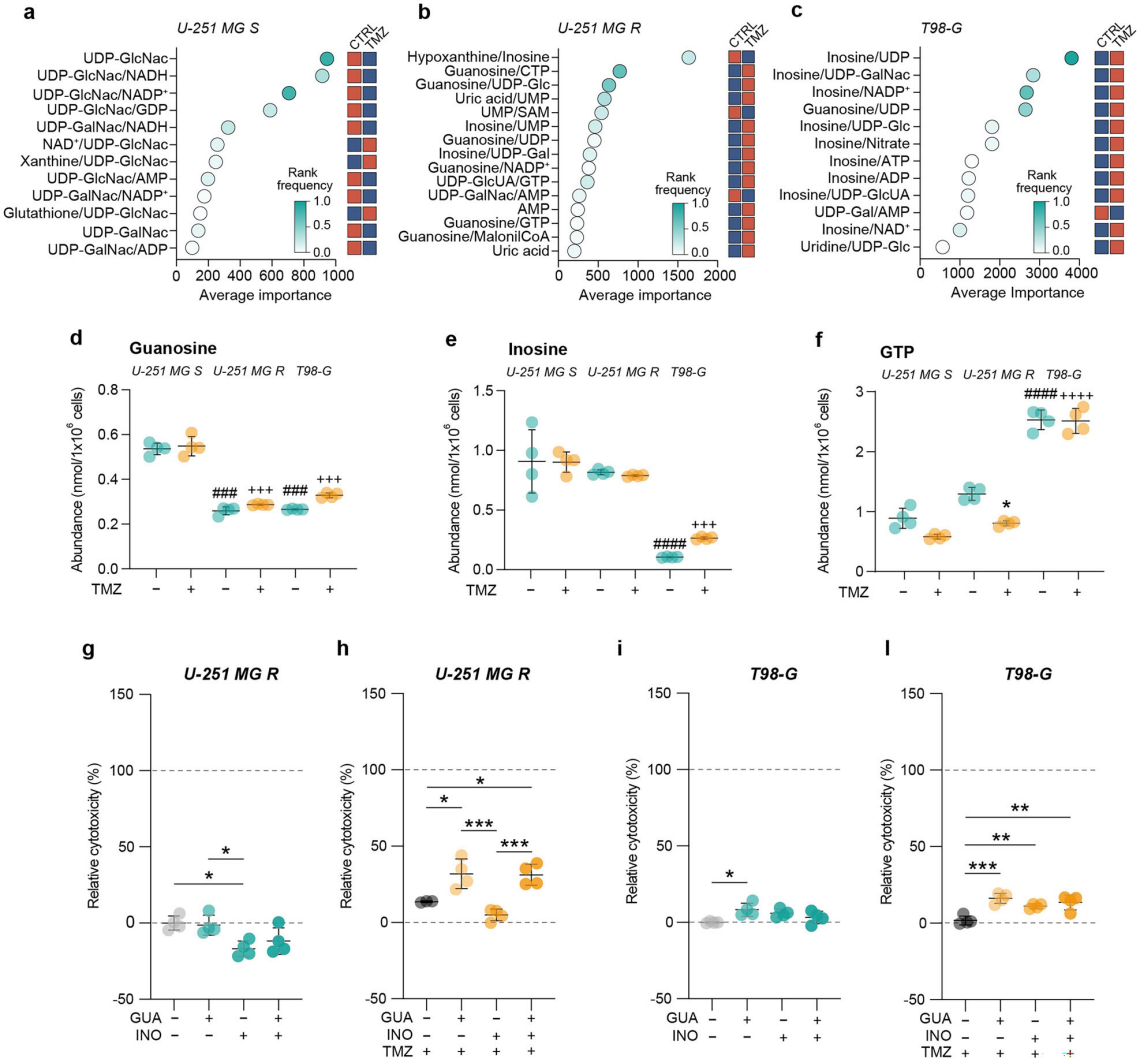
Guanosine and inosine are involved in TMZ resistance. **a**–**c** Metabolite ratio analysis plots, representing ratios with higher average importance for U-251 MG S (**a**), U-251 MG R (**b**) and T98-G (**c**). Blue squares indicate lower levels or ratios between metabolites and red squares indicate higher levels or ratios between metabolites in CTRL *vs* TMZ. **d** Guanosine abundance (expressed as nmol/1 × 10^6^ cells) in CTRL versus TMZ-treated cells for U-251 MG S, U-251 MG R and T98-G. **e** Inosine abundance (expressed as nmol/1 × 10^6^ cells) in CTRL versus TMZ-treated cells for U-251 MG S, U-251 MG R and T98-G. **f** GTP abundance (expressed as nmol/1 × 10^6^ cells) in CTRL versus TMZ-treated cells for U-251 MG S, U-251 MG R and T98-G. Data are shown via scattered dot plots as mean ± SD of n = 4 independent experiments. **g** LDH assay at 24 h on U-251 MG R, CTRL or treated with guanosine and/or inosine. **h** LDH assay at 24 h on U-251 MG R, treated with TMZ and/or guanosine/inosine. **i** LDH assay at 24 h on T98-G, CTRL or treated with guanosine and/or inosine. **l** LDH assay at 24 h on T98-G, treated with TMZ and/or guanosine/inosine. Data are shown via scattered dot plots as mean ± SD of n ≥ 3 independent experiments. *p-value < 0.05; **p-value < 0.01; ***p-value < 0.001 vs untreated or between groups; ###p-value < 0.001; ####p-value < 0.0001 vs untreated U-251 MG S; +++p-value < 0.001; ++++p-value < 0.0001 vs TMZ treated U-251 MG S. CTRL control, GUA guanosine, INO inosine, TMZ temozolomide.

From metabolomics data, we performed a metabolite ratio analysis, which calculates the most significant ratios according to their rank frequency and average importance (Figs. 3a–c and S2). These findings highlight the metabolites that play a significant role in response to TMZ treatment in the specific cell line. Ratios with higher importance included UDP N-acetylglucosamine (UDP-GlcNac) for U-251 MG S cells, and, guanosine and inosine for U-251 MG R and T98-G cells, respectively (Fig. 3a–c). Analysing guanosine and inosine abundances from metabolomic analysis, we found that guanosine basal levels were significantly lower in both resistant cells as compared to the sensitive ones, and that inosine levels were reduced in T98-G *vs* U-251 MG cell lines (Fig. 3d, e). Vice versa, T98-G cells exhibited a higher abundance of GTP as compared to U-251 MG S and U-251 MG R cells (Fig. 3f). This evidence suggests that purine metabolism could play a key role in TMZ resistance.

We then performed an LDH assay to test the effects of purine metabolites, namely exogenous guanosine and inosine, on resistant cells. We exposed U-251 MG R and T98-G cells to exogenous guanosine and inosine treatment, also analyzing their combination with TMZ (Fig. 3g–l). A very slight effect on cytotoxicity was observed in the two cell lines treated with guanosine or inosine (Fig. 3g, i). Interestingly, exogenous increase of purine levels in resistant-induced cells U-251 MG R led to a significant increase in cytotoxicity mediated by TMZ (Fig. 3h). Data from T98-G cells treated with both purines co-administered with TMZ confirmed an increased relative cytotoxicity (Fig. 3l). These findings confirmed the importance of purines in chemotherapy resistance, showing that guanosine and inosine can increase sensitivity to TMZ in resistant cells.

### Mesenchymal-like GBM zebrafish model exhibits an upregulation of purine biosynthesis, while concurrently downregulating purine catabolism

**Fig. 4:**
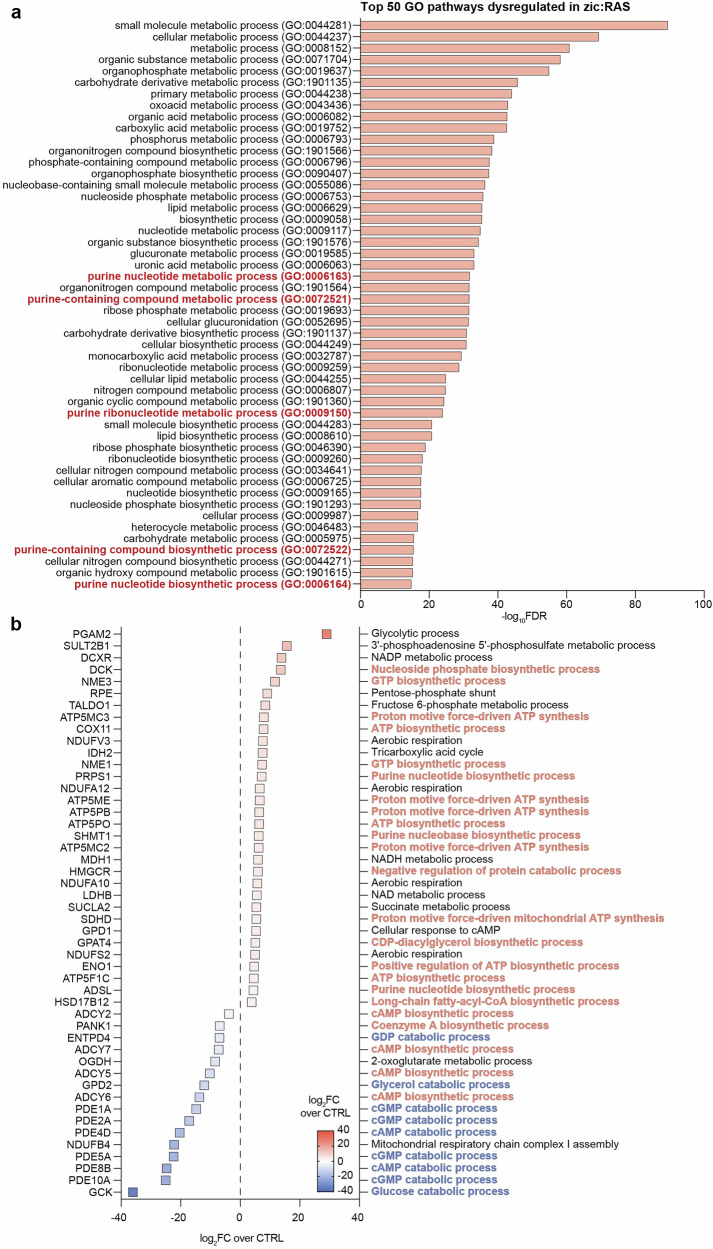
GBM zebrafish model shows increased purine biosynthesis and reduced purine catabolism. **a** RNA-seq analysis of the top 50 GO pathways dysregulated in the zic:RAS zebrafish model. Data are shown as −log_10_ of FDR. **b** Summary plot of genes involved in purine dysregulated pathways showed in (**a**) with the description of their functions. In red are represented functions correlated with biosynthetic processes and in blue functions correlated with catabolic processes. Squares are key-coloured according to their log_2_ FC over CTRL. CTRL control, FC fold change, FDR false discovery rate, GO gene ontology.

To examine the role of purine metabolism in GBM resistance, we employed a zebrafish GBM model, zic:RAS. Specifically, this model was developed through the expression of specific oncogenes during development in neural cells; indeed, somatic expression of oncogenic RAS spontaneously leads to GBM development, showing a mesenchymal-like profile [21]. On the zebrafish zic:RAS model, an RNA-seq and a Gene Ontology (GO) analysis of dysregulated pathways were performed. Our data demonstrated that about 10% of the top 50 dysregulated pathways were correlated to purine metabolism (Fig. 4a). Moreover, we evaluated genes involved in these pathways, revealing that most of the upregulated genes were implicated in purine biosynthetic mechanisms, whereas the downregulated genes were correlated to purine catabolic processes (Fig. 4b). These data demonstrated that altered purine metabolism represents a signature of mesenchymal GBM tumours, which typically exhibit resistance to TMZ, and that catabolic processes leading to guanosine and inosine accumulation are strongly reduced.

### Guanosine and inosine lead to alterations in mitochondrial dynamics

**Fig. 5:**
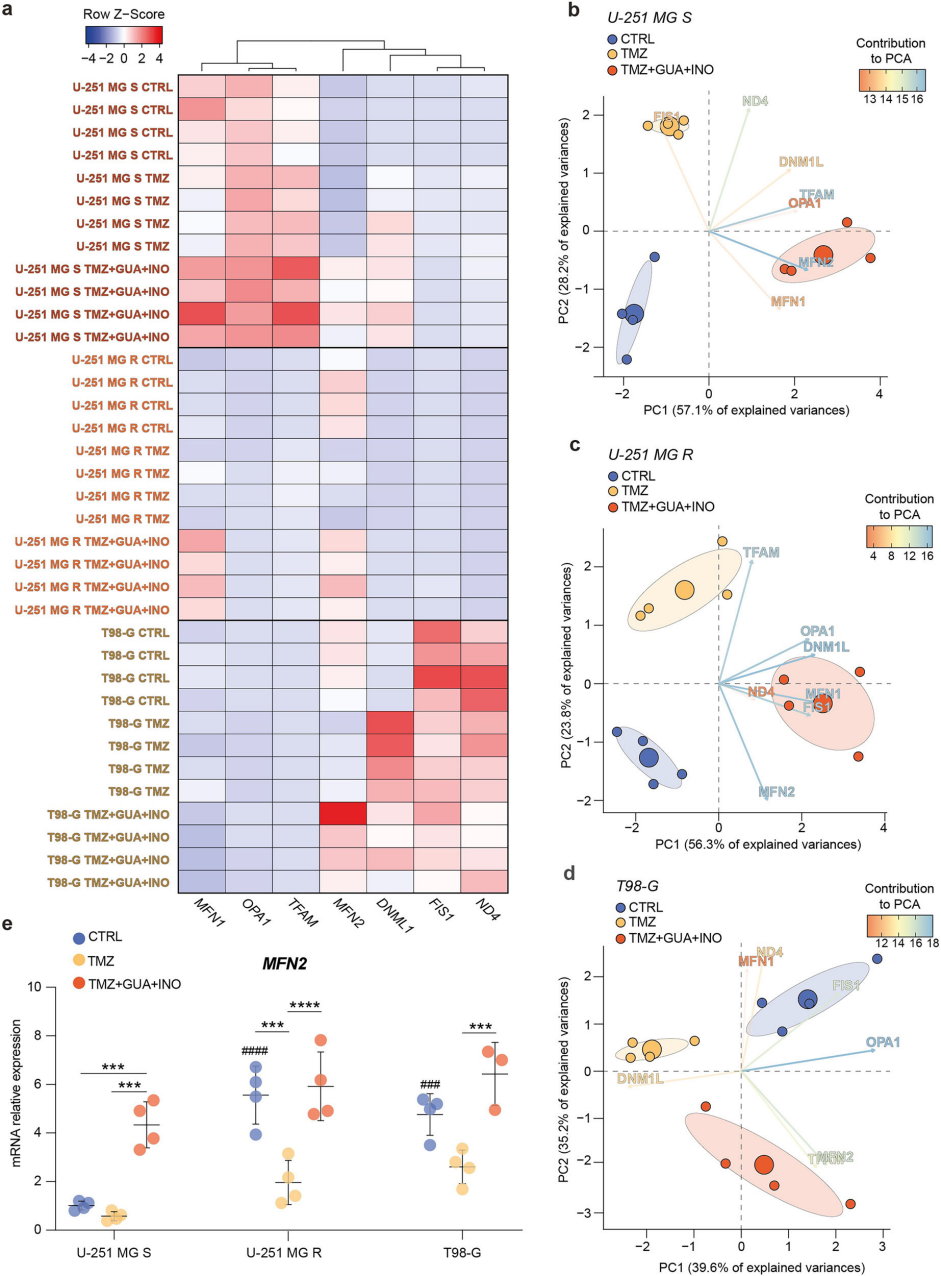
Guanosine and inosine increase MFN2 expression. **a** Heatmap of Z-Score values based on mRNA relative expression of 7 genes, involved in mitochondrial fusion and fission. **b**–**d** PCA biplot of 7 genes in CTRL, TMZ-treated and TMZ + GUA + INO-treated cells for U-251 MG S (**b**), U-251 MG R (**c**) and T98-G (**d**). Key-coloured arrows represent the contribution of each variable to the PCA; small circles are the single sample, while large circles are the mean points of the confidence ellipses. **e** mRNA expression levels of MFN2 in CTRL, TMZ-treated and TMZ + GUA + INO-treated cells for U-251 MG S, U-251 MG R, T98-G. Data are shown via scattered dot plots as mean ± SD of n ≥ 3 independent experiments. ***p-value < 0.001 and ****p-value < 0.0001 between groups. ###p-value < 0.001 and ####p-value < 0.0001 vs CTRL U-251 MG S. CTRL control, GUA guanosine, INO inosine, PCs principal components, PCA principal components analysis, TMZ temozolomide.

Our subsequent objective was to understand how purine levels could influence mitochondrial dynamics, investigating guanosine and inosine’s role in mitochondrial fitness. To this end, we first performed a qRT-PCR to evaluate on U-251 MG S, U-251 MG R and T98-G cells, mRNA expression levels of the main genes involved in mitochondrial fusion and fission. Data revealed a significant differential expression of tested genes among cell lines, identifying common clusters between U-251 MG R and T98-G cells, and distinct cluster for U-251 MG S cells (Fig. 5a).

We then performed a PCA analysis on qRT-PCR data to identify the highest contributors to the clustering between groups (Figs. S3 and 5b–d). Our data identified that Mitofusin-2 (MFN2) is a relevant factor in the group treated with TMZ combined with guanosine and inosine across all 3 tested cell lines (Fig. 5b–d). Indeed, purine metabolites in combination with TMZ increased MFN2 expression in both sensitive and resistant cells, improving mitochondrial fusion (Fig. 5e).

**Fig. 6:**
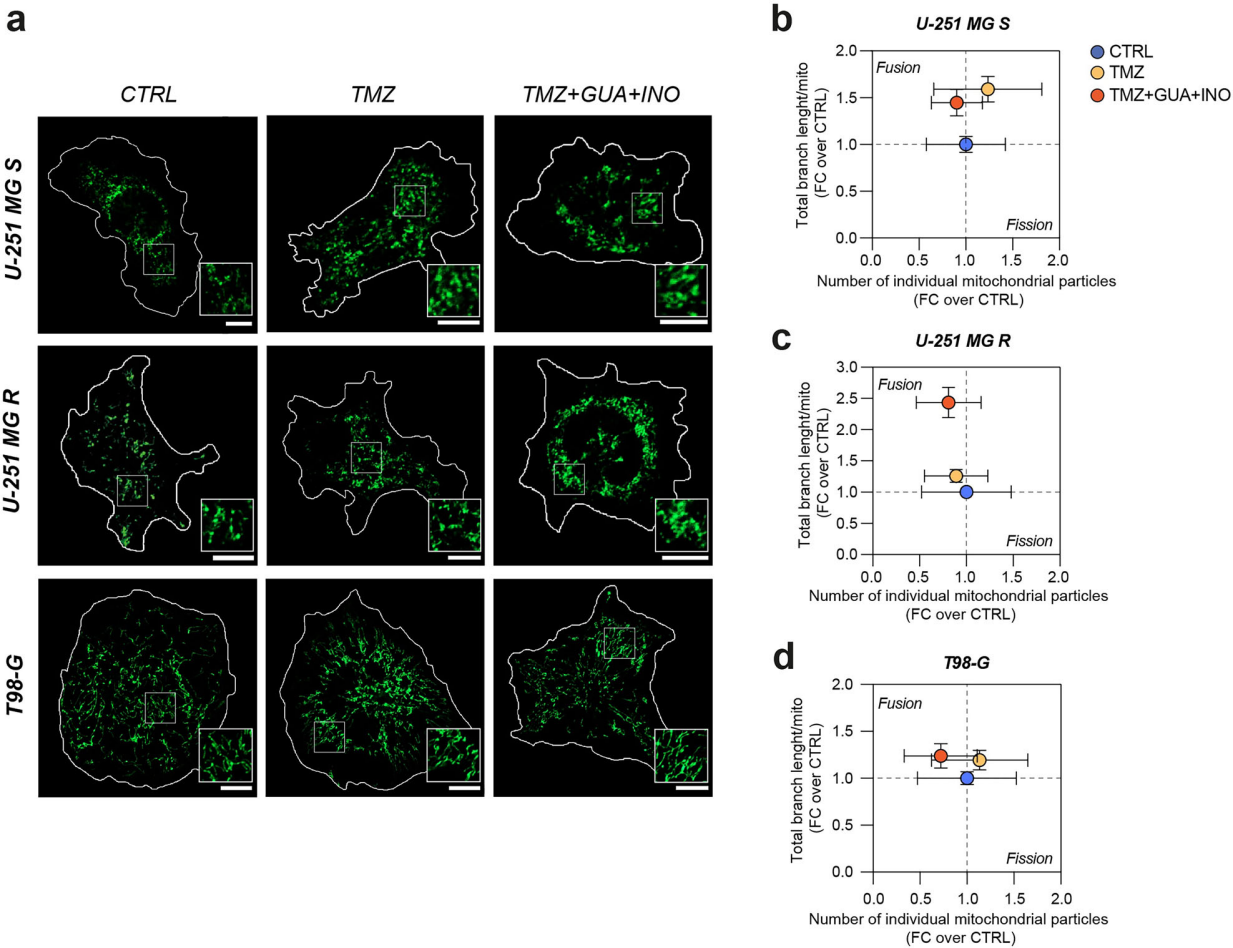
Guanosine and inosine, in combination with TMZ, stimulate mitochondrial fusion. **a** Representative micrographs of MitoView staining in CTRL, TMZ-treated and TMZ + GUA + INO-treated cells for U-251 MG S, U-251 MG R, T98-G. Scale bar = 10 μm. **b**–**d** Correlated quantification between total branch length/mito and number of individual mitochondrial particles in FC over CTRL for U-251 MG S (**b**), U-251 MG R (**c**) and T98- G (**d**). For U-251 MG S, CTRL: n = 37 cells from 4 biological replicates; TMZ: n = 33 cells from 4 biological replicates; TMZ + GUA + INO: n = 37 cells from 4 biological replicates. For U-251 MG R, CTRL: n = 26 cells from 4 biological replicates; TMZ: n = 45 cells from 4 biological replicates; TMZ + GUA + INO: n = 34 cells from 4 biological replicates. For T98-G, CTRL: n = 39 cells from 4 biological replicates; TMZ: n = 46 cells from 4 biological replicates; TMZ + GUA + INO: n = 40 cells from 4 biological replicates. Data are shown via scatter plots as mean ± SD. CTRL control, FC fold change, GUA guanosine, INO inosine, TMZ temozolomide.

In order to confirm mitochondrial fusion in cells treated with TMZ combined with guanosine and inosine, we performed a MitoView staining to analyse mitochondrial morphology and dynamics (Fig. 6a). We observed that the total branch length/mito increased in all tested cell lines as compared to control, while at the same time we observed a reduction in the number of individual mitochondrial particles in response to purines and TMZ treatment (Fig. 6b–d). These results support the hypothesis that guanosine and inosine enhance mitochondrial fusion. Moreover, we found changes in the number of branches and branch junctions as a result of treatment with TMZ combined with guanosine and inosine in both sensitive and resistant cells, confirming alterations in mitochondrial dynamics (Fig. S4).

**Fig. 7:**
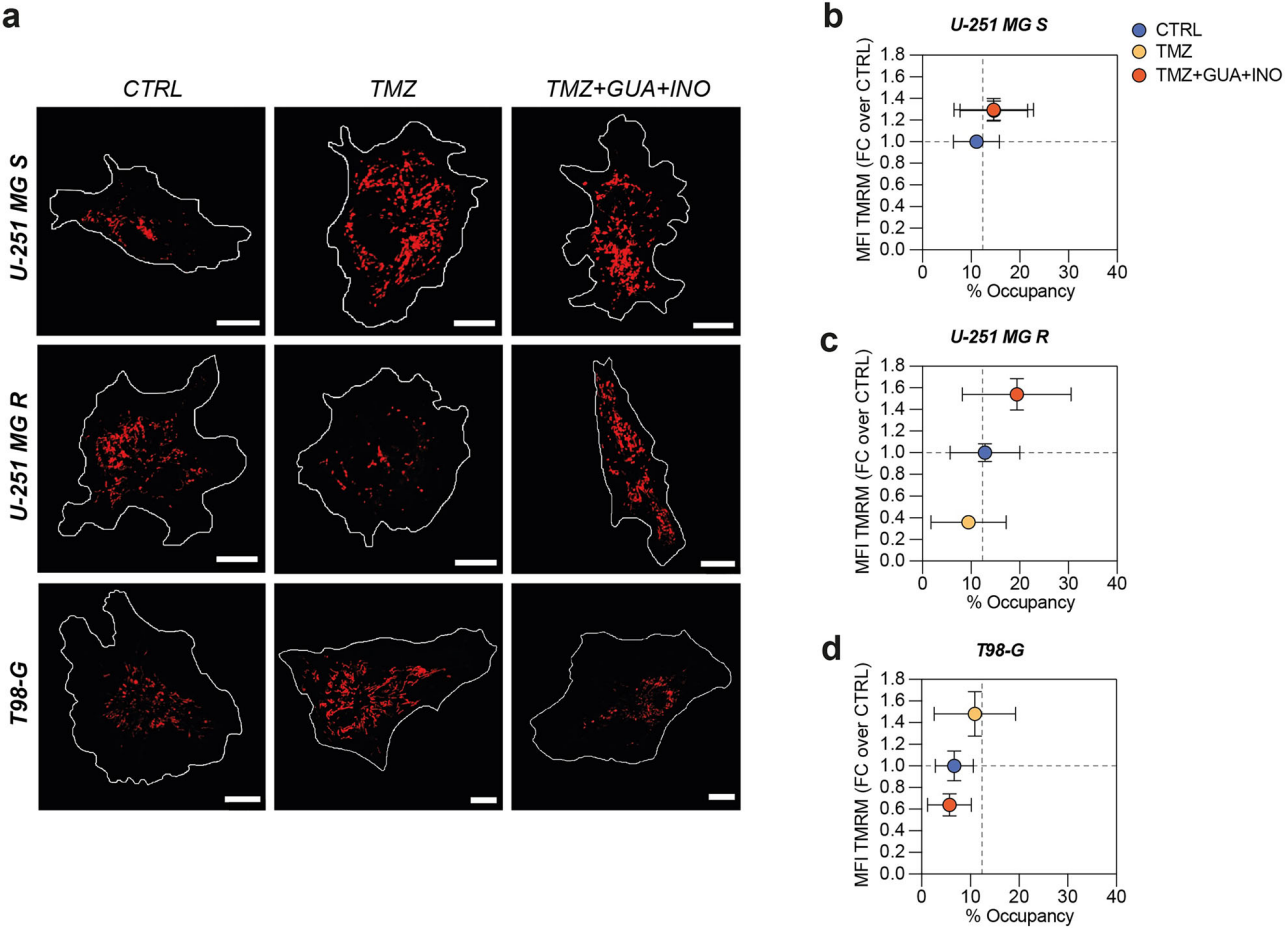
Resistant cells treated with TMZ, guanosine and inosine reshape their mitochondrial activity. **a** Representative micrographs of TMRM staining in CTRL, TMZ-treated and TMZ + GUA + INO-treated cells for U-251 MG S, U-251 MG R, T98-G. Scale bar = 10 μm. **b**–**d** Correlated quantification between MFI TMRM in FC over CTRL and % of occupancy for U-251 MG S (**b**), U-251 MG R (**c**) and T98- G (**d**). For U-251 MG S, CTRL: n = 49 cells from 4 biological replicates; TMZ: n = 46 cells from 4 biological replicates; TMZ + GUA + INO: n = 45 cells from 4 biological replicates. For U-251 MG R, CTRL: n = 46 cells from 4 biological replicates; TMZ: n = 33 cells from 4 biological replicates; TMZ + GUA + INO: n = 38 cells from 4 biological replicates. For T98-G, CTRL: n = 25 cells from 4 biological replicates; TMZ: n = 31 cells from 4 biological replicates; TMZ + GUA + INO: n = 27 cells from 4 biological replicates. Data are shown via scatter plots as mean ± SD. CTRL control, FC fold change, GUA guanosine, INO inosine, MFI mean fluorescence intensity, TMZ temozolomide.

Subsequently, we conducted a TMRM staining to quantify mitochondrial membrane potential (Δψm), which reflects mitochondrial activity (Fig. 7a–d). Our results showed that mitochondrial activity in U-251 MG S cells was not influenced by purine metabolites, coupled with an increase in MFI TMRM and in the percentage of occupancy after TMZ treatment that was maintained in cells treated with TMZ combined with guanosine and inosine (Fig. 7b).

However, purine metabolites strongly impacted mitochondrial activity in resistant cells (U-251 MG R and T98-G), reverting TMZ effect (Fig. 7c, d). Indeed, TMZ decreased MFI TMRM in U-251 MG R, but purine metabolites modified TMZ outcome, increasing both MFI TMRM and the percentage of occupancy (Fig. 7c). In contrast, T98-G cells improved mitochondrial activity following TMZ, reverting these effects after purine metabolites treatment (Fig. 7d). This evidence suggests that increasing purine metabolites, namely guanosine and inosine, may interfere with metabolic reprogramming employed by resistant cells to limit cytotoxic effects mediated by TMZ.

## Discussion

Metabolic reprogramming is one of the emerging mechanisms employed by GBM to enhance its aggressiveness and to better tolerate toxic effects of therapeutic treatments [22]. While glucose and lipid metabolic rewiring has been extensively investigated, studies on other metabolic pathways remain limited and most of the underlying mechanisms are not yet fully clarified [15]. Furthermore, it is well established that TMZ resistant cells alter different metabolic pathways to elude chemotherapeutic-induced damages and the subsequent cell death [23]. Thus, our aim was to analyze metabolic differences between TMZ-sensitive and resistant cells, identifying potential targets to increase chemotherapy efficacy. We selected two GBM cell lines: U-251 MG, which are TMZ-responsive, and T98-G, resistant to the drug [24]. In addition, we subjected U-251 MG cells to increasing concentrations of the chemotherapeutic agent to generate a third cell line, with morphological and mechanistic features similar to sensitive cells, but resistant to TMZ such as T98-G cells. Metabolomic analysis on these three cell lines revealed different levels of purine metabolites. Specifically, U-251 MG R and T98-G cells showed reduced guanosine levels as compared to U-251 MG S, while inosine was decreased only in mesenchymal cells, that on the contrary exhibited higher GTP levels. These results support the hypothesis that purine metabolic reprogramming is a mechanism involved in TMZ resistance. Recent evidence demonstrated that purine metabolism regulates radiotherapy resistance, revealing that GBM patients overexpressing enzymes implicated in the de novo GTP synthesis exhibited a shorter survival time [25]. Therefore, the inhibition of GTP synthesis reinforced radiation effects, increasing sensitivity of orthotopic GBM mouse model to radiotherapy [25]. Thus, to prove that guanosine and inosine could have effects on chemotherapy efficacy, we exposed resistant cells to purine metabolites in combination with TMZ, confirming their role in enhancing treatment effectiveness. The combination of guanosine and inosine with TMZ was effective in improving chemotherapy efficacy in both cell lines. However, considering the individual effects of the external purines, for U-251MG R cells, only guanosine was effective in combination with TMZ, while for T98-G cells, both guanosine and inosine were toxic when associated with the chemotherapeutic agent. These results could be related to the basal levels of guanosine and inosine in these two cell lines. Inosine levels in U-251 R cells were comparable to those in U-251 MG S, while T98-G cells exhibited significantly reduced levels of this metabolite. In contrast, guanosine basal levels were relatively low in both resistant cell lines as compared to the sensitive ones.

Oliveira et al. (2017) demonstrated that guanosine promoted cytotoxicity in TMZ-treated A172 GBM cells, inducing cell death through adenosine receptors [26]. Moreover, to confirm the role of purine metabolism in TMZ resistance, we performed an RNA-seq on a GBM zebrafish model, exhibiting a mesenchymal-like profile. The zic:RAS GBM zebrafish model is developed through the expression of specific oncogenes triggering mitogen-activated protein kinase (MAPK) and phosphoinositide 3-kinase (PI3K) signalling pathways in neural progenitor cells. Particularly, HRAS^V12^ induces invasive brain tumours, showing a gene signature overlapping with that of the GBM mesenchymal subtype [21]. RNA-seq data revealed that zic:RAS upregulates purine biosynthetic processes, while downregulates purine catabolic mechanisms. This evidence is in accordance with reduced abundance of guanosine and inosine and increased levels of GTP, deriving from nucleotide biosynthetic processes, in T98-G cells, exhibiting a mesenchymal profile [27]. Shireman et al. (2021) proved a correlation between de novo purine biosynthesis and chemoresistance; indeed, mycophenolate mofetil, blocking the activity of inosine monophosphate dehydrogenase 2 (IMPDH2) that is a rate-limiting enzyme of purine biosynthesis, increases TMZ efficacy in an in vivo GBM model [28]. We also demonstrated the role of catabolic products from purine metabolism and their involvement in TMZ resistance, thus not only de novo purine biosynthesis represents a driver of chemoresistance, but purine catabolism plays a crucial role too. Notably, increased TMZ susceptibility could be linked to the salvage pathway, an alternative process to synthetise purines by recycling purine bases or nucleosides, thereby producing new nucleotides with lower energy consumption [29]. GBM cells prefer de novo purine synthesis and downregulate salvage pathway. This mechanism may be related to the therapeutic effect of TMZ, which alkylates purines; thus, GBM cells may avoid recycling damaged purines upon TMZ treatment by limiting purine salvage pathway [28]. Adding guanosine and inosine could promote the salvage pathway to maintain the nucleotides pool; however, the exposure of GBM cells to purine metabolites in combination with TMZ, could lead to increased damaged purines recycling, resulting in an enhanced cytotoxicity for GBM cells.

Furthermore, our results support the treatment with purine and TMZ as modulators of mitochondrial dynamics, upregulating MFN2 and thus increasing mitochondrial fusion. MFN2 is an outer mitochondrial membrane GTPase and is pivotal in inducing mitochondrial fusion [30]. MFN2 role in cancer is still controversial, acting either as an onco-suppressor or oncogenic factor, depending on the tumour type and the tissue; however, in many solid tumours, MFN2 shows an anticancer function, inducing cell apoptosis and inhibiting proliferation through Bax-mediated apoptosis [31, 32]. Indeed, MFN2 overexpression, along with Drp1 knockdown, increases apoptosis in lung cancer cells, reducing their proliferation [33]. Recent studies revealed an unbalanced mitochondrial dynamic typical of tumour cells with hyperactive mitochondrial fission and impaired fusion [34]. GBM cells also exhibit higher rates of mitochondrial fission, showing a correlation with poor prognosis for patients. Indeed, increased mitochondrial fission and impaired fusion are correlated with enhanced tumour aggressiveness [35]. Therefore, this evidence suggests mitochondrial dynamics as a possible therapeutic target for GBM [36].

Our data revealed that guanosine and inosine can increase MFN2 expression, reverting TMZ effect that, on the contrary, reduces its expression in resistant cells. Thus, MFN2 downregulation could be a resistance to limit TMZ toxic effects, while purine metabolites revert this outcome. Moreover, mitochondrial fusion after purine metabolites treatment is confirmed by MitoView staining, in which we observed an increase in total branch length and a decrease in the number of individual mitochondrial particles as compared to control in all tested cell lines, further pointing towards an enhanced mitochondrial fusion [37]. Beyond alterations in dynamics, purine metabolites lead to changes in mitochondrial membrane potential, strongly correlated to mitochondrial functionality. In particular, while no differences in mitochondrial activity were observed in U-251 MG S cells exposed to TMZ alone or in combination with guanosine and inosine, resistant cells were strongly affected by TMZ combined with guanosine and inosine. Thus, a mechanism by which resistant cells counterbalance TMZ-induced cytotoxicity is related to reprogramming of their mitochondrial metabolism. This evidence is in accordance with previous literature demonstrating that tumours adapt their metabolism to evade drug cytotoxicity via mitochondrial metabolism reshaping, supporting their proliferation under stress conditions [38].

In conclusion, our data demonstrated that TMZ-sensitive and resistant cells show different metabolic profiles, shedding light on the role of purine metabolism in chemotherapy resistance. We observed in both in vitro and in vivo models an upregulation in purine biosynthetic processes and a decrease in purine catabolism in mesenchymal GBM, with significantly reduced guanosine and inosine, and increased GTP levels in GBM resistant cells. Moreover, purine metabolites treatment improves TMZ efficacy in resistant cells, increasing cytotoxicity and altering mitochondrial dynamics. Guanosine and inosine stimulate mitochondrial fusion upregulating MFN2 and revert TMZ-induced effects on mitochondrial membrane potential. Therefore, purine metabolism plays a crucial role in metabolic rewiring associated with TMZ resistance. Finally, guanosine and inosine could be considered as adjuvant treatments to increase chemotherapy effectiveness in resistant GBM tumours.

## Materials and methods

### Cell lines culture

Experiments were performed on U-251 MG (RRID: CVCL_0021) and T98-G (RRID: CVCL_0556) human GBM cell lines. Cells were purchased from the European Collection of Authenticated Cell Cultures (ECACC, Public Health England). U-251 MG and T98-G were cultured in growth medium consisting of Dulbecco’s Modified Eagle Medium (DMEM) High glucose (Cat#11965092, Gibco, Grand Island, NY, USA) supplemented with 10% Foetal Serum Bovine (FBS, Cat#26140079, Gibco), 100 IU/mL Penicillin-Streptomycin solution (pen-strep, Cat#15140-122, Gibco) and 1 mmol/L sodium pyruvate (Cat#11360-039, Gibco). Cells were maintained in an incubator at 37 °C with 95% air and 5% CO_2_ and sub-cultured in standard culture flasks.

### Temozolomide resistance-induced cells

U-251 MG cells, naturally sensitive (S) to TMZ (U-251 MG S), were subjected to increasing concentrations of TMZ (Cat#T2577, Sigma-Aldrich, Darmstadt, Germany) to create a resistant-induced cell line [39]. Briefly, in the first round, cells were cultured in a growth medium containing 20 μM TMZ for the first passage, followed by a wash-out phase of 3 passages without TMZ. In the second round, cells were treated with 40 μM TMZ for 3 consecutive passages, and, in the third round, U-251 MG were subjected to 60 μM TMZ for 3 additional consecutive passages, reaching TMZ resistance. These cells were then tested for TMZ resistance and named U-251 MG R.

### Lactate dehydrogenase assay

Lactate dehydrogenase (LDH) activity assay (Cat#CBA-241, Cell Biolabs, Inc., San Diego, CA, USA) was assessed to measure the relative cytotoxicity after treatments. Cells were seeded at a final density of 1 × 10^4^ cells/well in 96-well plates. After 24 h, cells were subjected to different sets of treatments for 24, 48 or 72 h (as stated in figure legends): (i) vehicle (i.e. DMSO and/or PBS); (ii) TMZ at different concentrations (50, 100, 250, 500 μM); (iii) 500 μM Guanosine (Cat#G6264, Sigma-Aldrich); (iv) 500 μM Inosine (Cat#I4125, Sigma-Aldrich); (v) 250 μM Guanosine combined with 250 μM Inosine; (vi) 250 μM TMZ combined with 500 μM Guanosine; (vii) 250 μM TMZ combined with 500 μM Inosine (Cat#I4125, Sigma-Aldrich); (viii) 250 μM TMZ combined with 250 μM Guanosine and 250 μM Inosine. Control cells received an equal amount of vehicle (i.e. PBS and/or DMSO) as per treated cells. In LDH assay experiments, DMSO was used at a concentration equal to 1% of the total volume. Cells treated with 1% of triton X-100 solution (Cat#124102, Cell Biolabs, Inc.) were considered as positive controls (100% relative cytotoxicity). Vehicle-treated cells were used as negative controls (0% relative cytotoxicity). LDH activity quantification was assessed by evaluating supernatants, following the manufacturer’s instructions. The absorbance was measured at 450 nm using a Multiskan SkyHigh Microplate spectrophotometer (Thermo Scientific, Waltham, MA, USA). The percentage of relative cytotoxicity was calculated using the formula:

$$\% {relative\; cytotoxicity}=(\frac{{ODsample}-{ODnegative\; CTRL}}{{ODpositive\; CTRL}-{ODnegative\; CTRL}})* 100$$

### Clonogenic assay

Clonogenic assay was performed by seeding cells at low density 400 cells/well in a 6-well plate. Cells were treated with vehicle (DMSO) or TMZ at a final concentration of 50 μM, for 24 or 72 h. After 10 days, colonies were fixed with methanol (Cat#412381, Carlo Erba, Milan, Italy) for 15 min at room temperature. Then, colonies were stained with crystal violet (Cat#61135, Sigma-Aldrich) for 25 min at room temperature. A colony composed of more than 50 cells were considered as a clone. Plating efficiency (PE) of controls was calculated as:

$$P.E.\quad =\frac{{Number\; of\; clones}}{{Number\; of\; plated\; cells}}$$

The percentage of surviving fraction (SF) was calculated as:

$$S.F.\quad ( \% {over\; CTRL})=\left(\frac{P.E.sample}{P.E.CTRL}\right)* 100$$

The average size was quantified in Fiji (version 2.14.0/1.54j).

### Targeted metabolomics and metabolite ratio analysis

For analysis of metabolites, cells were treated with vehicle (DMSO) or 50 µM TMZ for 24 h and then trypsinized, resuspended in PBS (1 × 10^6^ cells/ml) and centrifuged at 300 × *g* for 5 min at room temperature. Cellular pellet was deproteinized through a precipitating solution (75% acetonitrile + 25% KH_2_PO_4_, 10 mM, pH 7.4) and centrifuged at 20,890 × *g* for 10 min at 4 °C. Afterwards, supernatant was supplemented with chloroform to extract the aqueous phase. Separation of selected metabolites was achieved by flowing samples through a C18 chromatographic column (Hypersil C-18, 250 × 4.6 mm, 5 μm particle size) settled in a HPLC apparatus (ThermoFisher Scientific, Spectra System P4000 pump). The diode array detector UV6000 (ThermoFisher Scientific) tuned at wavelength of both 206 nm and 260 nm was used for metabolites identification and quantification.

Employing metabolomics data, metabolite ratio analysis was performed through MetaboAnalyst 5.0. Top ratios were calculated, based on the average importance and rank frequency, for all tested cell lines.

### Zebrafish model

Adult zebrafish (Danio rerio) were housed in the Model Organism Facility–Center for Integrative Biology (CIBIO) University of Trento and maintained under standard conditions. All zebrafish studies were performed according to European and Italian law, D.Lgs. 26/2014, authorisation 148/2018-PR to M. C. Mione. Fishes which developed brain tumours, called zic:RAS, were generated as described [21]. The following zebrafish transgenic lines were used during this study to generate the GBM model:

- Et(zic4:Gal4TA4, UAS:mCherry)_hzm5_ called zic:Gal4 [21];
- Tg(UAS:eGFP-HRAS_G12V)_io006_ called UAS:RAS [40].

### RNA-seq

RNA, library preparation and sequencing were done as previously described for 3 control brains (from 3 month old zic:Gal4 fish) [21] and 3 tumour brains (from 3 month old zic:RAS fish) [41]. Demultiplexed raw reads (fastq) generated from the Illumina HiSeq were checked using FASTQC tool (version 0.11.3) where all the samples passed the quality standards. Alignment was done against the reference genome Danio rerio assembly GRCz10 using STAR with recommended options and thresholds (version 2.5) HTSeq-count (version 0.9.1) was used to generate raw gene counts. Counts normalisation to Trimmed Mean of M-values (TMM) for visualisation methods was performed by edgeR package (v.3.24.3). Analysis of the expressions of the genes involved in metabolism in zebrafish brain tumours was performed following the protocol previously described [42].

Specifically, we used 1421 human genes assigned to human metabolic pathways described in the Kyoto Encyclopedia of Genes and Genomes (KEGG) database [43]. Zebrafish orthologs of metabolic genes were retrieved using the DIOPT online tool (https://fgr.hms.harvard.edu/diopt) and filtered for a match score equal or greater than 6. The differential expression analysis was performed using DESeq2 package (v.1.16). Top 50 GO pathways were calculated through Gene Ontology and pathway analysis.

### qRT-PCR

Gene expression on U-251 MG and T98-G was tested by performing qRT-PCR. Cell pellets were resuspended in TRIzol (Cat#15596018, Thermo Fisher Scientific). RNA extraction was performed by chemical separation, while cDNA was obtained by using High-Capacity cDNA Reverse Transcription Kit (Cat#4368814, Thermo Fisher Scientific) according to manufacturer’s protocol. Gene expression was analyzed using PowerUp™ SYBR™ Green Master Mix for (Cat#A25741, Thermo Fisher Scientific) and Rotor-Gene Q 2plex (Qiagen, Hilden, Germany). The relative expression level was determined by comparison with the control housekeeping ribosomal RNA 18S by using the 2^−ΔΔCt^ method. Primers used for this assay are reported in Table 1.

**Table 1:** List of primers used for qRT-PCR.

Gene	Forward Primer	Reverse primer
ND4	ACAAGCTCCATCTGCCTACGACAA	TTATGAGAATGACTGCGCCGGTGA
FIS1	ACTACCGGCTCAAGGAATACG	CATGCCCACGAGTCCATCTT
DNML1	TGGAGGCGCTAATTCCTGTC	TCTGCTTCCACCCCATTTTCT
MFN2	GGGAAGGTGAAGCGCAA	TTGTCCCAGAGCATGGCATT
TFAM	CCGAGGTGGTTTTCATCTGT	AGTCTTCAGCTTTTCCTGCG
OPA1	GAAAGGAGCTCATCTGTTTGGAGTC	TTCTTCCGGAGAACCAAAATCG
MFN1	TCGGGAAGATGAGGCAGTTT	TGCCATTATGCTAAGTCTCCG
18S	CTTAGAGGGACAAGTGGCG	ACGCTGAGCCAGTCAGTGTA

### Principal component analysis

Principal component analysis (PCA) was performed on qRT-PCR data. Scree plot with explained variances and corr plot with principal components (PCs) contribution expressed as square cosine (cos^2^) are shown. PCA is expressed as a biplot of variables and key-coloured arrows represent each variable. Analyses were performed using the RStudio software (Version: 2024.09.1 + 394).

### MitoView and TMRM staining and analysis

Cells were plated on Poly-D-Lysine (PDL)-coated glass coverslips treated with vehicle or 50 μM TMZ or 50 μM TMZ combined with 250 μM Guanosine and 250 μM Inosine. After 24 h, cells were washed in extracellular solution (ECS) and incubated with 150 nM MitoView Green (Cat#70054-T, Biotium, San Francisco, CA, USA) for 30 min at 37 °C, then with 100 nM Tetramethylrhodamine, Methyl Ester, Perchlorate (TMRM) (Cat#T668, Invitrogen, Waltham, MA, USA) for 30 min at 37 °C, diluted in ECS.

Live images of MitoView Green and TMRM stained cells were acquired using a LSM 800 confocal microscope with a Plan-Apochromat 63×/1.4 oil DIC M27 objective (Carl Zeiss, Oberkochen, Germany) using 488 nm, and 561 nm diode laser excitation. Emission spectra were acquired sequentially with 505–555 nm (MitoView) and 555–700 nm (TMRM) bandpass emission filters. Image stack series of cells (500-nm-thick z stacks) were recorded.

Morphological analysis of mitochondria stained with MitoView was performed by the Mitochondria analyser plugin [44] run in Fiji [45] using Fiji (version 2.14.0/1.54j). To describe the morphology of mitochondria, the total number of branches, number of branches/mito and number of branches junctions/mito were calculated. TMRM mean fluorescence intensity (MFI) and % of occupancy were quantified with Fiji (version 2.14.0/1.54j), defining the area of each cell.

### Statistical analysis

Data analysis was performed using GraphPad Prism software version 8.0.1. The sample size for each experiment is reported in the figure legends. No statistical methods were used to predetermine sample sizes, but our sample sizes were similar to those reported in previous publications [21, 46]. Quantifications were performed by operators blinded to the treatment, and no data points or animals were excluded from the analysis ex-ante. Outliers were identified using ROUT method with a Q = 1%. The data were assessed for normality distribution by using the Shapiro–Wilk test or D’Agostino & Pearson test, according to the sample size, followed by an evaluation for homogeneity of variance. Datasets that passed both tests were analysed with a two-tailed unpaired Student’s t-test for comparison of n = 2 groups, or one-way or two-way analysis of variance (ANOVA), followed by Holm-Sidak post-hoc test for multiple comparisons, for comparison of n ≥ 3 groups. Datasets with non-normal distribution were analysed using Kruskal–Wallis test. Data are expressed as mean ± standard deviation (SD). For all statistical tests, p-values < 0.05 were considered statistically significant.

## Supplementary information

Supplementary material

## Data Availability

The datasets used and/or analysed during the current study are available from the corresponding on reasonable request.
