# Supplementary material for: Purine metabolism rewiring improves glioblastoma susceptibility to temozolomide treatment
Source: Cell Death Dis. 2025 Apr 24;16(1):336. doi: 10.1038/s41419-025-07667-0 (PMC12022057; doi:10.1038/s41419-025-07667-0)
Supplement: Supplementary file 1 — Supplementary material [file 41419_2025_7667_MOESM1_ESM.pdf]

Supplementary material

Purine metabolism rewiring improves glioblastoma susceptibility to temozolomide treatment

Simona D'Aprile[1], Simona Denaro[1], Filippo Torrisi[2,3], Lucia Longhitano[1], Sebastiano Giallongo[2], Cesarina Giallongo[4], Vittorio Bontempi[5], Claudio Bucolo[1], Filippo Drago[1], Maria Caterina Mione[5], Giovanni Li Volti[1], Maja Potokar[6,7], Jernej Jorgačevski[6,7], Robert Zorec[6,7], Daniele Tibullo[1], Angela Maria Amorini[1], Nunzio Vicario[1,*], Rosalba Parenti[1,*].

[1]Department of Biomedical and Biotechnological Sciences, University of Catania, Catania, Italy;
[2]Department of Medicine and Surgery, University of Enna "Kore", Enna, Italy;
[3]Department of Drug and Health Sciences, University of Catania, Catania, Italy;
[4]Department of Medical and Surgical Sciences and Advanced Technologies, F. Ingrassia, University of Catania, Catania, Italy;
[5]Department of Cellular, Computational and Integrative Biology (CIBIO) and Centre of Medical Sciences (CISMed), University of Trento, Trento, Italy;
[6]Laboratory of Neuroendocrinology-Molecular Cell Physiology, Institute of Pathophysiology, Faculty of Medicine, University of Ljubljana, Ljubljana, Slovenia
[7]Celica Biomedical, Ljubljana, Slovenia
*Correspondence: nunziovicario@unict.it; parenti@unict.it.

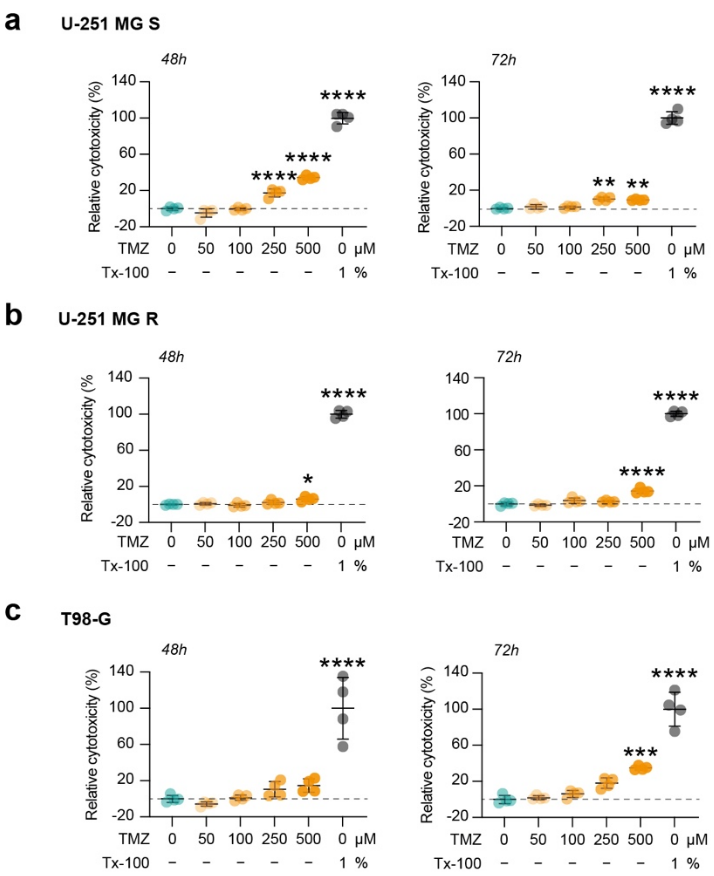

Supplementary figure 1. a-c) LDH assay at 48 h and at 72 h on U-251 MG S (a), U-251 MG R (b) and T98-G (c) cells treated with 0, 50, 100, 250, 500 µM of TMZ. Data are shown via scattered dot plots as mean ± SD of n = 4 independent experiments. *p-value < 0.05; **p-value < 0.01; *** p-value < 0.001; **** p-value < 0.0001.
CTRL, control; TMZ, temozolomide; Tx-100, Triton X-100.

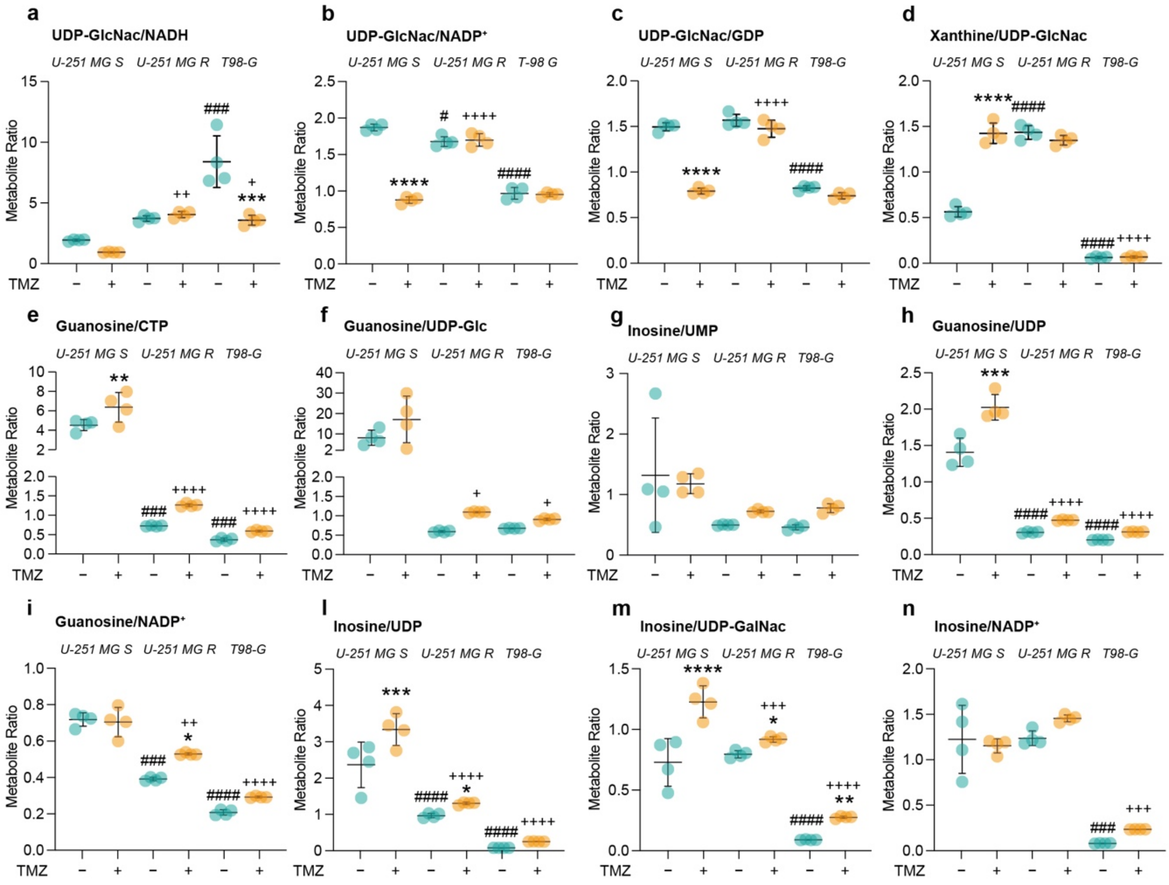

Supplementary figure 2. a-n) Quantification of the most important metabolite ratios, CTRL versus TMZ-treated cells for U-251 MG S, U-251 MG R and T98-G. * TMZ-treated versus untreated; # untreated versus untreated U-251 MG S; + TMZ-treated versus TMZ-treated U-251 MG S. Data are shown via scattered dot plots as mean ± SD of n=4 independent experiments. *p-value < 0.05; **p-value < 0.01; ***p-value < 0.001 and ****p-value < 0.0001 vs untreated. #p-value < 0.05; ###p-value < 0.001 and ####p-value < 0.0001 vs untreated U-251 MG S. +p-value < 0.05; ++p-value < 0.01; +++p-value < 0.001 and ++++p-value < 0.0001 vs TMZ treated U-251 MG S.

CTRL, control; TMZ, temozolomide.

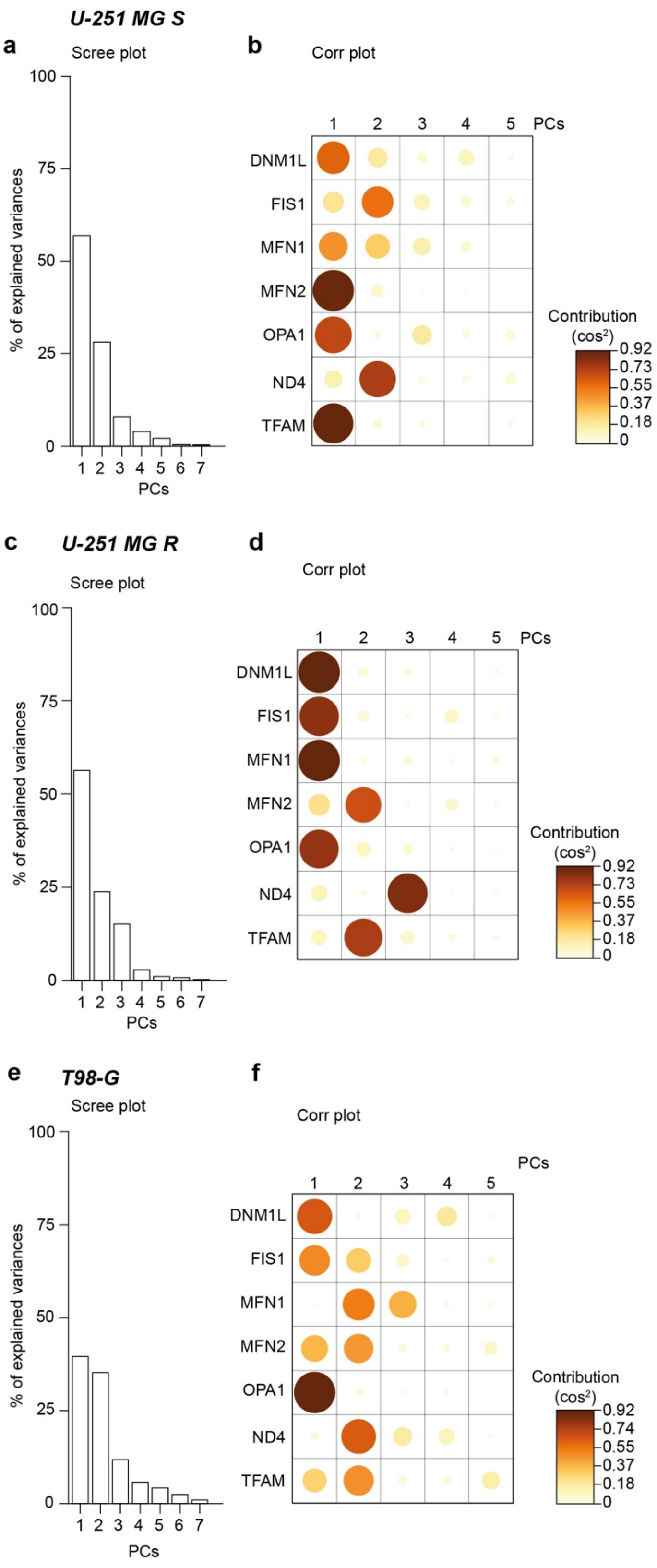

Supplementary figure 3. a, c, e) Scree plot for U-251 MG S (a), U-251 MG R (c) and T98-G (e) cells, showing % of explained variances for each PCs. b, d, f) Corr plot for U-251 MG S (b), U-251 MG R (d) and T98-G (f) cells, representing PC1-PC5 contributions expressed as $\cos^2$ for each gene. PCs, principal components; PCA, principal components analysis.

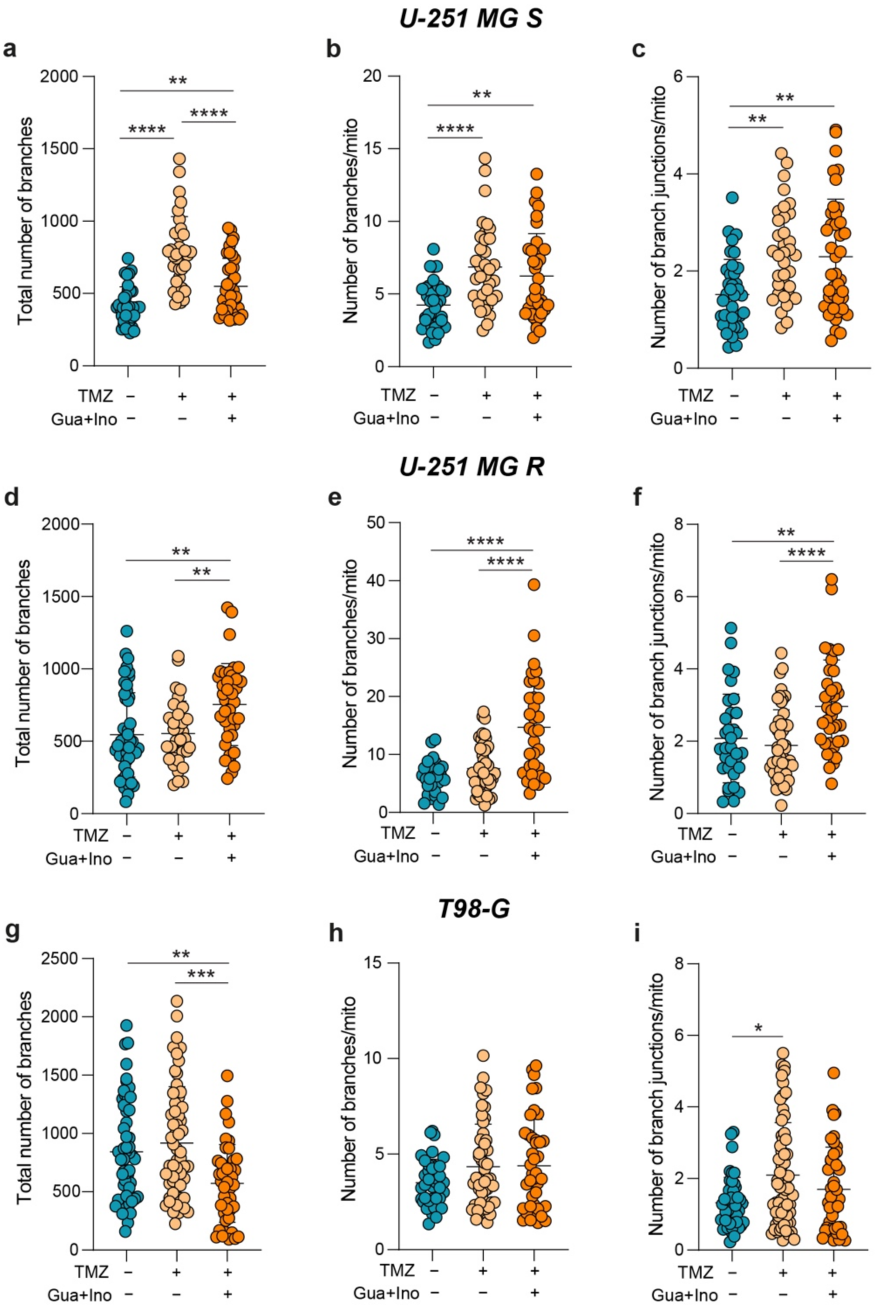

Supplementary figure 4. a, d, g) Quantification of the total number of branches in CTRL, TMZ-treated and TMZ+GUA+INO-treated cells for U-251 MG S (a), U-251 MG R (d) and T98-G (g). b, e, h) Quantification of number of branches/mito in CTRL, TMZ-treated and TMZ+GUA+INO-treated cells for U-251 MG S (b), U-251 MG R (e) and T98-G (h). c, f, i) Quantification of number of branch junctions/mito in CTRL, TMZ-treated and TMZ+GUA+INO-treated cells for U-251 MG S (c), U-251 MG R (f) and T98-G (i). Data are shown via scattered dot plots as mean ± SD. *p-value < 0.05; **p-value < 0.01; ***p-value < 0.001; ****p-value < 0.0001. GUA, guanosine; INO, inosine; TMZ, temozolomide.
